# Approximate solution of Newell-Whitehead-Segel model with time-fractional derivative

**DOI:** 10.1371/journal.pone.0288740

**Published:** 2023-07-20

**Authors:** Jinxing Liu, Muhammad Nadeem, Yahya Alsayyad

**Affiliations:** 1 Faculty of Science, Yibin University, Yibin, China; 2 School of Mathematics and Statistics, Qujing Normal University, Qujing, China; 3 Department of Physics, Hodeidah University, Al-Hudaydah, Yemen; University of Porto Faculty of Engineering: Universidade do Porto Faculdade de Engenharia, PORTUGAL

## Abstract

In the current analysis, we developed a significant approach for deriving the approximate solution of the Newell-Whitehead-Segel model with Caputo derivatives. This scheme is developed based on Sumudu transform and the residual power series method (RPSM) that generates the solution in the form of a series. First, we apply the Sumudu transform to decompose the fractional order and obtain a recurrence relation. Secondly, we utilize the RPSM to the recalescence relation and then we can derive the series solution with successive iterations using the initial conditions. We observe that this approach demonstrates a high accuracy and validity to the proposed fractional model. In our developed scheme, we do not face any huge calculation and restriction of elements that diverse the significance of the results. In addition, we display 2D and 3D graphical visuals to show the physical nature of the fractional model.

## 1 Introduction

Fractional calculus (FC) is a generalization of integer-order calculus to arbitrary-order calculus that was created during the closure of the 17th century. The fundamental benefit of FC is that it explains an extremely valuable strategy for studying the memory and hereditary features in a wide range of phenomena. In addition, ordinary calculus is also a subset of FC. Over the last few decades, the fundamental study of fractional derivatives has been improved very quickly. The study of FC has been applied to various fields such as electrodynamics, the study of chaos, and optical science [1–3]. Numerous scholars and scientists have contributed to the significance of FC in different branches of sciences such as fractional-order two-dimensional Helmholtz equations [4], nonlinear coupled fractional massive thirring equation [5], Klein-Gordon equation [6], time-fractional Navier-Stokes equation [7], nonlinear shock wave equations with fractional order [8], fractional Fokas Lenells equation [9], fractional acoustic waves model [10], Korteweg-de Vries (KdV) equation [11], fractional study of the non-linear burgers’ equations [12] and many other nonlinear system such as high order uncertain nonlinear systems [13], nonlinear networked control systems [14], radial basis function neural network model [15], Bayesian network analysis [16], reaction-diffusion system [17] and Keller-Segel model [18]. Finding the exact solution of these nonlinear fractal models is still challenging thus analytical and numerical schemes are the most suitable approaches to derive the fractal solution of these nonlinear models. Finding the exact solution of these nonlinear fractional models is still challenging thus analytical and numerical schemes are the most suitable approaches to derive the fractal solution of these nonlinear models.

Consider the classical two-dimensional Newell-Whitehead-Segel (NWS) model such as

$$\begin{array}{c} \vartheta_{t}=\vartheta_{xx}+a_{1}\vartheta -a_{2}\vartheta^{l},\;0\;<\alpha <\;1, \end{array}$$ (1)

where *ϑ*(*x*, *t*) shows the function with space *x* and time *t*. *a*_1_, *a*_2_ > 0 and *r* is a positive integer. In Eq (1), the initial element on the left section represents the variation of *ϑ*(*x*, *t*) with respect to time at a specific place and similarly the initial element on the right section shows the variations of *ϑ*(*x*, *t*) with variable *x* at time *t* whereas *a*_1_*ϑ* − *a*_2_*ϑ*^*r*^ symbolizes a known source. Let us convert the Eq (1) into time-fractional NWS form such as:

$$\begin{array}{c} D_{t}^{\alpha }\vartheta \left(x,t\right)=\vartheta_{xx}+a_{1}\vartheta -a_{2}\vartheta^{l},\;0\;<\alpha <\;1, \end{array}$$ (2)

where *α* represents the Caputo fractional derivative. The classic NWS is one of the famous amplitude problems for predicting the presence of stationary spatial stripe configurations in a two-dimensional system [19, 20]. Two different patterns may be seen: the hexagonal pattern, where the honeycomb and striped cells are made by splitting the liquid flow, and the roll pattern, where the cylinders are developed using fluid streamlines that may be bent and form spiral-like patterns. In particular, zebra skin, the visual brain, and human fingerprints all have striped patterns. It is important to note that the laser beam propagation across nonlinear optical medium, hexagonal patterns can be produced in a chemical reaction and diffusion model [21]. Saravanan and Magesh [22] provided the schemes of reduced differential transform and obtained the analytical solution of the NWS model. Luo and Nadeem [23] proposed a combined strategy of Laplace transform with the residual power series to derive the approximate solution of the NWS model with fractional order. Patade and Bhalekar [24] presented the idea of the Laplace Adomian transform scheme to achieve the numerical solution of the NWS model. Saadeh et al. [25] proposed the idea of a fractional residual power series algorithm to obtain the results of the NWS equation with time-fractional order. Nadeem et al. [26] developed a scheme, called the Natural homotopy perturbation method for obtaining the approximate solution of the fractional Newell-Whitehead-Segel problem. Besides this, various analytical and numerical schemes [27–29] have serious flaws in their development such as assumption theory and constraints on elements, convolution theorem, and integration that are challengeable tasks to derive the solution in series form.

This paper highlights the development of SRPSM, based on the combined form of ST and the RPSM for the approximate solution of NWS model with Caputo derivatives. This scheme provides the results in the form of a series that moves towards the precise results. RPSM can handle both linear and nonlinear problems. Some graphical representations have been shown to explain the physical nature of the fractional NWS model. The 2D and 3D visuals express the robustness of our proposed strategy. The error distribution demonstrates the nature of fractional order. This scheme does not require any theory of assumption and restricting of variables. We design this article such as; we present a few concepts of FC and ST in Section (2). We developed the idea of SRPSM in Section (3). In Section (4), we illustrate two numerical applications to show the authenticity of our proposed strategy and eventually, we explain the conclusion in Section (5).

## 2 Concepts of fractional calculus and Sumudu transform

In this part, we will go through the fundamental concepts of fractional calculus and the Sumudu transform.

**Definition 2.1** Let *ϑ*(*t*) ∈ *C*_*μ*_, *μ* ≥ 1 be a function, then the Riemann-Liouville operator of fractional order *α* > 1 is defined as [30, 31]

$$\begin{array}{c} J^{\alpha }\vartheta \left(t\right)=\frac{1}{\Gamma (\alpha )}\int_{0}^{t}{\left(t-\tau \right)}^{\alpha -1}\vartheta \left(\tau \right)d\tau ,\left(\alpha >0\right)\\ J^{0}\vartheta \left(t\right)=\vartheta \left(t\right). \end{array}$$

Also, we have

$$\begin{array}{c} J^{\alpha }t^{y}=\frac{\Gamma (y+1)}{\Gamma (y+\alpha +1)}t^{\alpha +y}. \end{array}$$

**Definition 2.2** The fractional derivative of *ϑ*(*t*) in the Caputo sense is expressed as [32]:

$$\begin{array}{c} D_{t}^{\alpha }\vartheta \left(t\right)=J^{k-\alpha }D^{n}\;f\left(t\right)=\frac{1}{\Gamma (n-\alpha )}\int_{0}^{1}{\left(t-\tau \right)}^{k-\alpha -1}\vartheta^{k}\left(\tau \right)d\tau , \end{array}$$

for k-1<α≤k,k∈N,t>0.

**Definition 2.3** The ST is expressed as follows [33]

$$\begin{array}{c} A=\{\vartheta \left(t\right)|\exists M,\tau_{1},\tau_{2}>0,|f\left(t\right)|<Me^{\left|t\right|/\tau_{j}},\;\text{if}\;t\in {\left(-1\right)}^{j}\times \left[0,\infty \right)\} \end{array}$$

in the following form

$$\begin{array}{c} S\left[\vartheta \left(t\right)\right]=\frac{1}{q}\int_{0}^{\infty }\vartheta \left(t\right)e^{-\frac{t}{q}}dt,\;0<\kappa_{1}\leq q\leq \kappa_{2}. \end{array}$$

where S is called the ST operator.

**Definition 2.4** The ST of the Caputo fractional derivative is explained as [34]:

$$\begin{array}{c} S[D_{t}^{\alpha }\vartheta \left(t\right)]=r^{-\alpha }S\left[\vartheta \left(t\right)\right]-\sum \;r^{-\alpha +\kappa }\vartheta^{\kappa }\left(0\right).\;\kappa -1<\alpha \leq \kappa \end{array}$$

**Definition 2.5** Let the series [35]

$$\begin{array}{c} \sum_{n=0}^{\infty }\;\vartheta_{n}{\left(t-t_{0}\right)}^{n\alpha }=\vartheta_{0}\left(x\right)+\vartheta_{1}\left(x\right){\left(t-t_{0}\right)}^{\alpha }+\vartheta_{2}\left(x\right){\left(t-t_{0}\right)}^{2\alpha }+\cdots ,\;0<n-1<\alpha \leq n,\;t\geq t_{0} \end{array}$$

is defined as the power series about *t* = *t*_0_, where *t* is a variable and *x* is called as the coefficients of the series.

## 3 Development of Sumudu residual power series method (SRPSM)

This section presents the development of the Sumudu residual power series method to obtain the approximate results of the NWS model with fractal derivatives. We straightforwardly build this concept and explain it step by step. Consider the nonlinear fractional model such as

$$\begin{array}{c} D_{t}^{\alpha }\vartheta \left(x,t\right)=L\vartheta \left(x,t\right)+N\vartheta \left(x,t\right)+g\left(x,t\right), \end{array}$$ (3)

with the initial condition

$$\begin{array}{c} \vartheta (x,t)=a(x), \end{array}$$ (4)

**Step 1**: Using ST on Eq (3), we get

$$\begin{array}{c} S[D_{t}^{\alpha }\vartheta \left(x,t\right)]=S[L\vartheta (x,t)+N\vartheta (x,t)+g(x,t)]. \end{array}$$

Applying the property of ST along the initial condition (4), we get

$$\begin{array}{c} S\left[\vartheta \left(x,t\right)\right]=a\left(x\right)+r^{\alpha }[L\vartheta (x,t)+N\vartheta (x,t)+g(x,t)]. \end{array}$$ (5)

**Step 2**: Employing the inverse ST on Eq (5), it yields

$$\begin{array}{c} \vartheta \left(x,t\right)=G\left(x,t\right)+S^{-1}[r^{\alpha }\{L\vartheta (x,t)+N\vartheta (x,t)\}], \end{array}$$ (6)

where

$$\begin{array}{c} G\left(x,t\right)=a\left(x\right)+S^{-1}[r^{\alpha }g\left(x,t\right)]. \end{array}$$

**Step 3**: We use the classic RPSM, the algorithm can be proposed by

$$\begin{array}{c} \vartheta \left(x,t\right)=\sum_{n=0}^{\infty }\;\vartheta_{n}\left(x\right)\frac{t^{n\alpha }}{\Gamma (n\alpha +1)}. \end{array}$$ (7)

Now, we propose the *n*-the series of *ϑ*(*x*, *t*) to derive the approximate solution of Eq (7) in such a way,

$$\begin{array}{c} \vartheta_{n}\left(x,t\right)=\sum_{n=0}^{k}\vartheta_{n}\left(x\right)\frac{t^{n\alpha }}{\Gamma (n\alpha +1)}. \end{array}$$ (8)

**Step 4**: Now, the residual function for Eq (8) is expressed as

$$\begin{array}{c} \text{Res}\;\vartheta_{n}\left(x,t\right)=\vartheta_{n}\left(x,t\right)-[G\left(x,t\right)+S^{-1}\{r^{\alpha }(L\vartheta_{n-1}\left(x,t\right)+N\vartheta_{n-1}\left(x,t\right))\}]. \end{array}$$ (9)

Thus, the *n*-th residual function yields such that

$$\begin{array}{c} \text{Res}\;\vartheta_{n}\left(x,t\right)\;{|}_{t=0}=0, \end{array}$$ (10)

so we have

$$\begin{array}{cc} \vartheta_{n}\left(x,t\right) & =\vartheta_{0}\left(x,t\right)+\vartheta_{1}\left(x,t\right)+\vartheta_{2}\left(x,t\right)+\vartheta_{3}\left(x,t\right)+\cdots ,\\ \vartheta (x,t) & =\underset{N\to 0}{\text{lim}}\;\sum_{n=0}^{N}\vartheta_{n}\left(x,t\right), \end{array}$$

where

$$\begin{array}{cc} \vartheta_{0}\left(x,t\right) & =\vartheta_{0}\left(x,t\right),\\ \vartheta_{1}\left(x,t\right) & =\vartheta_{1}\left(x,t\right)\frac{t^{\alpha }}{\Gamma (1+\alpha )},\\ \vartheta_{2}\left(x,t\right) & =\vartheta_{2}\left(x,t\right)\frac{t^{2\alpha }}{\Gamma (1+2\alpha )},\\ \vdots \\ \vartheta_{n}\left(x,t\right) & =\vartheta_{n}\left(x,t\right)\frac{t^{n\alpha }}{\Gamma (1+n\alpha )}. \end{array}$$

## 4 Numerical applications

In this part, we illustrate two numerical applications of the fractional NWS model for checking the performance of SRPSM. Our derived solution showed a high accuracy toward the precise results in just a couple of iterations. This scheme has the advantage of fewer calculations as compared to the described schemes in the literature and yields the results in terms of series. We also demonstrate the 2D and 3D graphical structures and use the Mathematical software 11 to display the physical nature of the fractional NWS model.

### 4.1 Example 1

Consider *a*_1_ = −2, *a*_2_ = 0, *l* = 1, the model of Eq (2) tends to the following problem such as

$$\begin{array}{c} \frac{\partial^{\alpha }\vartheta }{\partial t^{\alpha }}=\vartheta_{xx}-2\vartheta , \end{array}$$ (11)

with initial conditions

$$\begin{array}{c} \vartheta \left(x,0\right)=e^{x}. \end{array}$$ (12)

Taking the ST, we get

$$\begin{array}{c} S[\frac{\partial^{\alpha }\vartheta }{\partial t^{\alpha }}]=S[\frac{\partial^{2}\vartheta }{\partial x^{2}}-2\vartheta ]. \end{array}$$

By means of of ST, we get,

$$\begin{array}{c} S[\vartheta (x,t)]=\vartheta \left(x,0\right)+r^{\alpha }S[\frac{\partial^{2}\vartheta }{\partial x^{2}}-2\vartheta ]. \end{array}$$ (13)

Applying inverse ST, Eq (13) yields

$$\begin{array}{c} \vartheta \left(x,t\right)=\vartheta \left(x,0\right)+S^{-1}[r^{\alpha }S\{\frac{\partial^{2}\vartheta }{\partial x^{2}}-2\vartheta \}]. \end{array}$$ (14)

Now, we have the RPSM such as

$$\begin{array}{c} \vartheta_{n}\left(x,t\right)=\sum_{n=0}^{k}\vartheta_{n}\left(x\right)\frac{t^{n\alpha }}{\Gamma (n\alpha +1)}. \end{array}$$ (15)

Using the strategy of SRPSM, Eq (13) yields as follows

$$\begin{array}{c} \text{Res}\;\vartheta_{n}\left(x,t\right)=\vartheta_{n}\left(x,t\right)-\vartheta \left(x,0\right)-S^{-1}[r^{\alpha }S\{\frac{\partial^{2}\vartheta_{n-1}}{\partial x^{2}}-2\vartheta_{n-1}\}], \end{array}$$ (16)

when *n* = 0, we obtain

$$\begin{array}{c} \text{Res}\;\vartheta_{0}\left(x,t\right)=\vartheta_{0}\left(x,t\right)-\vartheta \left(x,0\right). \end{array}$$ (17)

But from Eq (15), we get

$$\begin{array}{c} \vartheta_{0}\left(x,t\right)=\vartheta_{0}\left(x\right). \end{array}$$

Since Res *ϑ*_0_(*x*, *t*) ∣_*t*=0_ = 0, thus Eq (17) yields

$$\begin{array}{c} \vartheta_{0}\left(x,t\right)=e^{x}. \end{array}$$ (18)

Now, to determine the value of *ϑ*_1_(*x*, *t*), Let *n* = 1 in Eq (16), we obtain

$$\begin{array}{c} \text{Res}\;\vartheta_{1}\left(x,t\right)=\vartheta_{1}\left(x,t\right)-\vartheta \left(x,0\right)-S^{-1}[r^{\alpha }S\{\frac{\partial^{2}\vartheta_{0}}{\partial x^{2}}-2\vartheta_{0}\}]. \end{array}$$ (19)

But from Eq (15), we get

$$\begin{array}{c} \vartheta_{1}\left(x,t\right)=\vartheta_{0}\left(x\right)+\vartheta_{1}\left(x\right)\frac{t^{\alpha }}{\Gamma (\alpha +1)}. \end{array}$$ (20)

From Eqs (19) and (20), we obtain as follows

$$\begin{array}{c} \text{Res}\;\vartheta_{1}\left(x,t\right)=\vartheta_{1}\left(x\right)\frac{t^{\alpha }}{\Gamma (\alpha +1)}-S^{-1}[r^{\alpha }S\{\frac{\partial^{2}\vartheta_{0}}{\partial x^{2}}-2\vartheta_{0}\}]. \end{array}$$

After the simplifications, we can obtain such as

$$\begin{array}{c} \text{Res}\;\vartheta_{1}\left(x,t\right)=\vartheta_{1}\left(x\right)\frac{t^{\alpha }}{\Gamma (\alpha +1)}+e^{x}\frac{t^{\alpha }}{\Gamma (\alpha +1)}. \end{array}$$ (21)

Since Res *ϑ*_1_(*x*, *t*) ∣_*t*=0_ = 0, thus Eq (21) yields

$$\begin{array}{c} \vartheta_{1}\left(x\right)=-e^{x}. \end{array}$$ (22)

Now, to determine the value of *ϑ*_2_(*x*, *t*), Let *n* = 2 in Eq (16), we obtain

$$\begin{array}{c} \text{Res}\;\vartheta_{2}\left(x,t\right)=\vartheta_{2}\left(x,t\right)-\vartheta \left(x,0\right)-S^{-1}[r^{\alpha }S\{\frac{\partial^{2}\vartheta_{1}}{\partial x^{2}}-2\vartheta_{1}\}]. \end{array}$$ (23)

But using Eq (15), we have

$$\begin{array}{c} \vartheta_{2}\left(x,t\right)=\vartheta_{0}\left(x\right)+\vartheta_{1}\left(x\right)\frac{t^{\alpha }}{\Gamma (\alpha +1)}+\vartheta_{2}\left(x\right)\frac{t^{2\alpha }}{\Gamma (2\alpha +1)}. \end{array}$$ (24)

From Eqs (23) and (24), we obtain as follows

$$\begin{array}{c} \text{Res}\;\vartheta_{2}\left(x,t\right)=\vartheta_{0}\left(x\right)+\vartheta_{1}\left(x\right)\frac{t^{\alpha }}{\Gamma (\alpha +1)}+\vartheta_{2}\left(x\right)\frac{t^{2\alpha }}{\Gamma (\alpha +1)}-S^{-1}[r^{\alpha }S\{\frac{\partial^{2}\vartheta_{1}}{\partial x^{2}}-2\vartheta_{1}\}]. \end{array}$$

After the simplifications, we can obtain such as

$$\begin{array}{c} \text{Res}\;\vartheta_{2}\left(x,t\right)=\vartheta_{1}\left(x\right)\frac{t^{\alpha }}{\Gamma (\alpha +1)}+\vartheta_{2}\left(x\right)\frac{t^{2\alpha }}{\Gamma (\alpha +1)}+e^{x}\frac{t^{\alpha }}{\Gamma (\alpha +1)}-e^{x}\frac{t^{2\alpha }}{\Gamma (\alpha +1)}. \end{array}$$ (25)

Since Res *ϑ*_2_(*x*, *t*) ∣_*t*=0_ = 0, thus Eq (25) yields

$$\begin{array}{c} \vartheta_{2}\left(x\right)=e^{x}. \end{array}$$ (26)

Now, to determine the value of *ϑ*_3_(*x*, *t*), Let *n* = 3 in Eq (16), we obtain

$$\begin{array}{c} \text{Res}\;\vartheta_{3}\left(x,t\right)=\vartheta_{3}\left(x,t\right)-\vartheta \left(x,0\right)-S^{-1}[r^{\alpha }S\{\frac{\partial^{2}\vartheta_{2}}{\partial x^{2}}-2\vartheta_{2}\}]. \end{array}$$ (27)

But using Eq (15), we have

$$\begin{array}{c} \vartheta_{3}\left(x,t\right)=\vartheta_{0}\left(x\right)+\vartheta_{1}\left(x\right)\frac{t^{\alpha }}{\Gamma (\alpha +1)}+\vartheta_{2}\left(x\right)\frac{t^{2\alpha }}{\Gamma (2\alpha +1)}+\vartheta_{3}\left(x\right)\frac{t^{3\alpha }}{\Gamma (3\alpha +1)}. \end{array}$$ (28)

From Eqs (27) and (28), we obtain as follows

$$\begin{array}{c} \text{Res}\;\vartheta_{3}\left(x,t\right)=\vartheta_{0}\left(x\right)+\vartheta_{1}\left(x\right)\frac{t^{\alpha }}{\Gamma (\alpha +1)}+\vartheta_{2}\left(x\right)\frac{t^{2\alpha }}{\Gamma (2\alpha +1)}+\vartheta_{3}\left(x\right)\frac{t^{3\alpha }}{\Gamma (3\alpha +1)}-S^{-1}[r^{\alpha }S\{\frac{\partial^{2}\vartheta_{2}}{\partial x^{2}}-2\vartheta_{2}\}]. \end{array}$$

After the simplifications, we can obtain such as

$$\begin{array}{c} \begin{array}{cc} \text{Res}\;\vartheta_{3}\left(x,t\right) & =\vartheta_{1}\left(x\right)\frac{t^{\alpha }}{\Gamma (\alpha +1)}+\vartheta_{2}\left(x\right)\frac{t^{2\alpha }}{\Gamma (\alpha +1)}+\vartheta_{3}\left(x\right)\frac{t^{3\alpha }}{\Gamma (3\alpha +1)}\\ & +\;e^{x}\frac{t^{\alpha }}{\Gamma (\alpha +1)}-e^{x}\frac{t^{2\alpha }}{\Gamma (2\alpha +1)}+e^{x}\frac{t^{3\alpha }}{\Gamma (3\alpha +1)}. \end{array} \end{array}$$ (29)

Since Res *ϑ*_3_(*x*, *t*) ∣_*t*=0_ = 0, thus Eq (29) yields

$$\begin{array}{c} \vartheta_{3}\left(x\right)=-e^{x}. \end{array}$$ (30)

Proceeding in the similar process, we can obtain the Eq (15) such as

$$\begin{array}{c} \begin{array}{cc} \vartheta (x,t) & =\vartheta_{0}\left(x\right)+\vartheta_{1}\left(x\right)\frac{t^{\alpha }}{\Gamma (\alpha +1)}+\vartheta_{2}\left(x\right)\frac{t^{2\alpha }}{\Gamma (2\alpha +1)}+\vartheta_{3}\left(x\right)\frac{t^{3\alpha }}{\Gamma (3\alpha +1)}+\cdots ,\\ & =e^{x}-e^{x}\frac{t^{\alpha }}{\Gamma (\alpha +1)}+e^{x}\frac{t^{2\alpha }}{\Gamma (2\alpha +1)}-e^{x}\frac{t^{3\alpha }}{\Gamma (3\alpha +1)}+\cdots . \end{array} \end{array}$$ (31)

The obtained series moves towards the exact solution at *α* = 1 as follows

$$\begin{array}{c} \vartheta \left(x,t\right)=e^{x-t}. \end{array}$$ (32)

**Fig 1:**
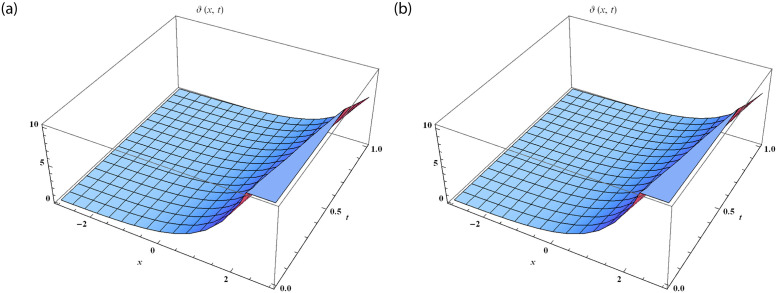
The 3D comparison of fractional NWS model with the SRPSM solution at *α* = 1 and the exact solution. (**a**) The approximate solution of *ϑ*_3_(*x*, *t*). (**b**) The exact solution of *ϑ*(*x*, *t*).

**Fig 2:**
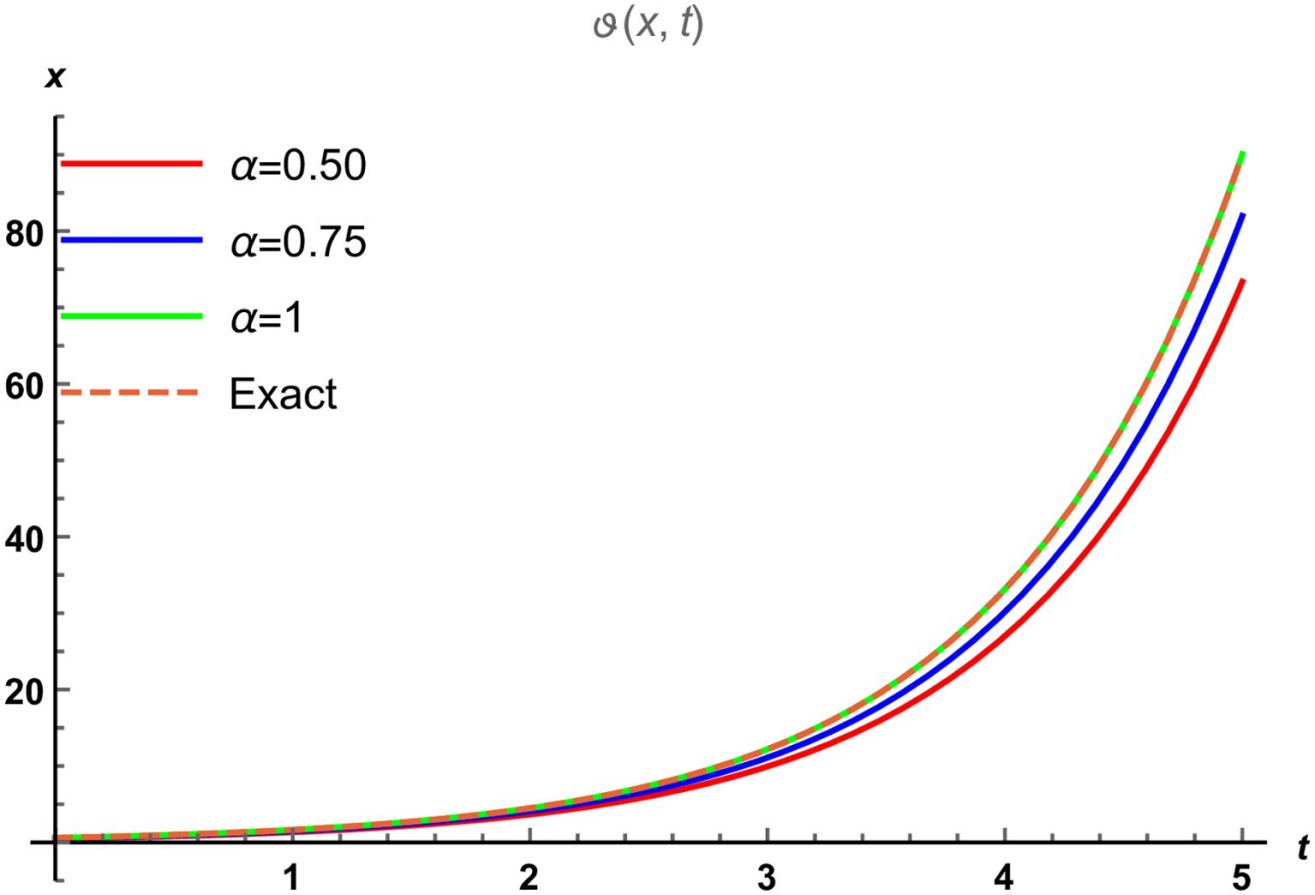
The physical nature of fractional NWS model compared with the exact solution and the SRPSM solution at various fractional order where *α* ∈ [0.5, 0.75, 1].

We divide the figures into two parts such that Fig 1a is designed by the solution of SRPSM at *α* = 1 and Fig 1b is designed by the exact solution of fractional NWS model. The range of space variable is taken as −3 ≤ *x* ≤ 3 at *t* = 1 for both 3D graphical solutions of Eqs (31) and (32). Fig 2 represents the physical nature of the fractional NWS model by considering the range of space values 0 ≤ *x* ≤ 5 and *t* = 0.5 at the various fractional order of *α* = 0.5, 0.75, 1 and then compared with the exact solution. We observe that the then compared with the exact solution. We observe that the approximate solution derived by SRPSM converges to the values. We consider our iteration only up to 3rd term and for better results these iterations can be extended. We also provide the absolute error in Table 1 to show that the approximate solutions obtained by our proposed strategy have excellent agreement with the exact solution. We provide these values at *α* = 0.50, 1 to show that the approximate solution becomes closer to the exact solution as the fractional order increases. The graphical structures and the absolute error display that our proposed strategy is valid and accurate.

**Table 1:** The error distribution between the results of SRPSM and the exact solution.

*x*	*t*	*ϑ*_3_(*x*, *t*) at *α* = 0.5	*ϑ*_3_(*x*, *t*) at *α* = 1	Exact solution *ϑ*(*x*, *t*)	Absolute Error |*ϑ*(*x*, *t*) − *ϑ*_3_(*x*, *t*)|
0.25	0.05	1.01345	1.2214	1.2214	0.0000
	0.15	0.859371	1.10514	1.10517	0.00003
	0.25	0.759859	0.999801	1.0000	0.000199
	0.35	0.676268	0.904088	0.904837	0.000749
	0.45	0.598328	0.81672	0.818731	0.002011
0.5	0.05	1.3013	1.56831	1.56831	0.0000
	0.15	1.10345	1.41903	1.41907	0.00004
	0.25	0.975678	1.28377	1.28403	0.00026
	0.35	0.868346	1.16087	1.16183	0.00096
	0.45	0.768268	1.04869	1.05127	0.00258
0.75	0.05	1.6709	2.01375	2.01375	0.00000
	0.15	1.41686	1.82208	1.82212	0.00004
	0.25	1.2528	1.64839	1.64872	0.00033
	0.35	1.11498	1.49059	1.49182	0.00123
	0.45	0.986476	1.34654	1.34986	0.00332
1	0.05	2.14548	2.58571	2.58571	0.0000
	0.15	1.81929	2.33959	2.33965	0.00006
	0.25	1.60862	2.11658	2.117	0.00042
	0.35	1.43166	1.91395	1.91554	0.00159
	0.45	1.26666	1.729	1.73325	0.00425

### 4.2 Example 2

Again, consider *a*_1_ = 1, *a*_2_ = 1 and *l* = 2, the model of Eq (2) tends to the following problem such as

$$\begin{array}{c} \frac{\partial^{\alpha }\vartheta }{\partial t^{\alpha }}=\vartheta_{xx}+\vartheta -\vartheta^{2}, \end{array}$$ (33)

with initial conditions

$$\begin{array}{c} \vartheta \left(x,0\right)=\frac{1}{{\left(1+e^{\frac{x}{\sqrt{6}}}\right)}^{2}}. \end{array}$$ (34)

Taking the ST, we get

$$\begin{array}{c} S[\frac{\partial^{\alpha }\vartheta }{\partial t^{\alpha }}]=S[\frac{\partial^{2}\vartheta }{\partial x^{2}}+\vartheta -\vartheta^{2}]. \end{array}$$

By means of of ST, we get,

$$\begin{array}{c} S[\vartheta (x,t)]=\vartheta \left(x,0\right)+r^{\alpha }S[\frac{\partial^{2}\vartheta }{\partial x^{2}}-\vartheta ]. \end{array}$$ (35)

Applying inverse ST, Eq (35) yields

$$\begin{array}{c} \vartheta \left(x,t\right)=\vartheta \left(x,0\right)+S^{-1}[r^{\alpha }S\{\frac{\partial^{2}\vartheta }{\partial x^{2}}+\vartheta -\vartheta^{2}\}]. \end{array}$$ (36)

Now, we have the RPSM such as

$$\begin{array}{c} \vartheta_{n}\left(x,t\right)=\sum_{n=0}^{k}\vartheta_{n}\left(x\right)\frac{t^{n\alpha }}{\Gamma (n\alpha +1)}, \end{array}$$ (37)

Using the strategy of SRPSM, Eq (36) yields as follows

$$\begin{array}{c} \text{Res}\;\vartheta_{n}\left(x,t\right)=\vartheta_{n}\left(x,t\right)-\vartheta \left(x,0\right)-S^{-1}[r^{\alpha }S\{\frac{\partial^{2}\vartheta_{n-1}}{\partial x^{2}}+\vartheta_{n-1}-\vartheta_{n-1}^{2}\}], \end{array}$$ (38)

when *n* = 0, we obtain

$$\begin{array}{c} \text{Res}\;\vartheta_{0}\left(x,t\right)=\vartheta_{0}\left(x,t\right)-\vartheta \left(x,0\right). \end{array}$$ (39)

But using Eq (37), we have

$$\begin{array}{c} \vartheta_{0}\left(x,t\right)=\vartheta_{0}\left(x\right). \end{array}$$

Since Res *ϑ*_0_(*x*, *t*) ∣_*t*=0_ = 0, thus Eq (39) yields,

$$\begin{array}{c} \vartheta_{0}\left(x,t\right)=\frac{1}{{\left(1+e^{\frac{x}{\sqrt{6}}}\right)}^{2}}. \end{array}$$ (40)

Now, to determine the value of *ϑ*_1_(*x*, *t*), Let *n* = 1 in Eq (38), we obtain

$$\begin{array}{c} \text{Res}\;\vartheta_{1}\left(x,t\right)=\vartheta_{1}\left(x,t\right)-\vartheta \left(x,0\right)-S^{-1}[r^{\alpha }S\{\frac{\partial^{2}\vartheta_{0}}{\partial x^{2}}+\vartheta_{0}-\vartheta_{0}^{2}\}]. \end{array}$$ (41)

But from Eq (37), we get

$$\begin{array}{c} \vartheta_{1}\left(x,t\right)=\vartheta_{0}\left(x\right)+\vartheta_{1}\left(x\right)\frac{t^{\alpha }}{\Gamma (\alpha +1)}. \end{array}$$ (42)

From Eqs (41) and (42), we obtain as follows

$$\begin{array}{c} \text{Res}\;\vartheta_{1}\left(x,t\right)=\vartheta_{0}\left(x\right)+\vartheta_{1}\left(x\right)\frac{t^{\alpha }}{\Gamma (\alpha +1)}-S^{-1}[r^{\alpha }S\{\frac{\partial^{2}\vartheta_{0}}{\partial x^{2}}+\vartheta_{0}-\vartheta_{0}^{2}\}]. \end{array}$$

After the simplifications, we can obtain such as

$$\begin{array}{c} \text{Res}\;\vartheta_{1}\left(x,t\right)=\vartheta_{1}\left(x\right)\frac{t^{\alpha }}{\Gamma (\alpha +1)}-\frac{5e^{\frac{x}{\sqrt{6}}}}{3{\left(1+e^{\frac{x}{\sqrt{6}}}\right)}^{3}}\frac{t^{\alpha }}{\Gamma (\alpha +1)}. \end{array}$$ (43)

Since Res *ϑ*_3_(*x*, *t*) ∣_*t*=0_ = 0, thus Eq (43) yields

$$\begin{array}{c} \vartheta_{1}\left(x\right)=\frac{5e^{\frac{x}{\sqrt{6}}}}{3{\left(1+e^{\frac{x}{\sqrt{6}}}\right)}^{3}}. \end{array}$$ (44)

Now, to determine the value of *ϑ*_2_(*x*, *t*), Let *n* = 2 in Eq (38), we obtain

$$\begin{array}{c} \text{Res}\;\vartheta_{2}\left(x,t\right)=\vartheta_{2}\left(x,t\right)-\vartheta \left(x,0\right)-S^{-1}[r^{\alpha }S\{\frac{\partial^{2}\vartheta_{1}}{\partial x^{2}}+\vartheta_{1}-\vartheta_{1}^{2}\}]. \end{array}$$ (45)

But using Eq (37), we have

$$\begin{array}{c} \vartheta_{2}\left(x,t\right)=\vartheta_{0}\left(x\right)+\vartheta_{1}\left(x\right)\frac{t^{\alpha }}{\Gamma (\alpha +1)}+\vartheta_{2}\left(x\right)\frac{t^{2\alpha }}{\Gamma (2\alpha +1)}. \end{array}$$ (46)

From Eqs (45) and (46), we obtain as follows

$$\begin{array}{cc} \text{Res}\;\vartheta_{2}\left(x,t\right) & =\vartheta_{1}\left(x\right)\frac{t^{\alpha }}{\Gamma (\alpha +1)}+\vartheta_{2}\left(x\right)\frac{t^{2\alpha }}{\Gamma (2\alpha +1)}-S^{-1}[r^{\alpha }S\{\frac{\partial^{2}}{\partial x^{2}}(\vartheta_{0}\left(x\right)+\vartheta_{1}\left(x\right)\frac{t^{\alpha }}{\Gamma (\alpha +1)})\\ & +(\vartheta_{0}\left(x\right)+\vartheta_{1}\left(x\right)\frac{t^{\alpha }}{\Gamma (\alpha +1)})-{\left(\vartheta_{0}\left(x\right)+\vartheta_{1}\left(x\right)\frac{t^{\alpha }}{\Gamma (\alpha +1)}\right)}^{2}\}]. \end{array}$$

After the simplifications, we can obtain such as

$$\begin{array}{c} \text{Res}\;\vartheta_{2}\left(x,t\right)=\vartheta_{2}\left(x\right)\frac{t^{2\alpha }}{\Gamma (2\alpha +1)}-\frac{25e^{\frac{x}{\sqrt{6}}}\left(-1+2e^{\frac{x}{\sqrt{6}}}\right)}{18{\left(1+e^{\frac{x}{\sqrt{6}}}\right)}^{4}}\frac{t^{2\alpha }}{\Gamma (2\alpha +1)}, \end{array}$$ (47)

Since Res *ϑ*_3_(*x*, *t*) ∣_*t*=0_ = 0, thus Eq (47) yields

$$\begin{array}{c} \vartheta_{2}\left(x\right)=\frac{25e^{\frac{x}{\sqrt{6}}}\left(-1+2e^{\frac{x}{\sqrt{6}}}\right)}{18{\left(1+e^{\frac{x}{\sqrt{6}}}\right)}^{4}}. \end{array}$$ (48)

Now, to determine the value of *ϑ*_3_(*x*, *t*), Let *n* = 3 in Eq (38), we obtain

$$\begin{array}{c} \text{Res}\;\vartheta_{3}\left(x,t\right)=\vartheta_{3}\left(x,t\right)-\vartheta \left(x,0\right)-S^{-1}[r^{\alpha }S\{\frac{\partial^{2}\vartheta_{2}}{\partial x^{2}}+\vartheta_{2}-\vartheta_{2}^{2}\}]. \end{array}$$ (49)

But using Eq (37), we have

$$\begin{array}{c} \vartheta_{3}\left(x,t\right)=\vartheta_{0}\left(x\right)+\vartheta_{1}\left(x\right)\frac{t^{\alpha }}{\Gamma (\alpha +1)}+\vartheta_{2}\left(x\right)\frac{t^{2\alpha }}{\Gamma (2\alpha +1)}+\vartheta_{3}\left(x\right)\frac{t^{3\alpha }}{\Gamma (3\alpha +1)}. \end{array}$$ (50)

From Eqs (49) and (50), we obtain as follows

$$\begin{array}{cc} \text{Res}\;\vartheta_{3}\left(x,t\right) & =\vartheta_{1}\left(x\right)\frac{t^{\alpha }}{\Gamma (\alpha +1)}+\vartheta_{2}\left(x\right)\frac{t^{2\alpha }}{\Gamma (2\alpha +1)}+\vartheta_{3}\left(x\right)\frac{t^{3\alpha }}{\Gamma (3\alpha +1)}\\ & -S^{-1}[r^{\alpha }S\{\frac{\partial^{2}}{\partial x^{2}}(\vartheta_{0}\left(x\right)+\vartheta_{1}\left(x\right)\frac{t^{\alpha }}{\Gamma (\alpha +1)}+\vartheta_{2}\left(x\right)\frac{t^{2\alpha }}{\Gamma (2\alpha +1)})\\ & +(\vartheta_{0}\left(x\right)+\vartheta_{1}\left(x\right)\frac{t^{\alpha }}{\Gamma (\alpha +1)}+\vartheta_{2}\left(x\right)\frac{t^{2\alpha }}{\Gamma (2\alpha +1)})-{\left(\vartheta_{0}\left(x\right)+\vartheta_{1}\left(x\right)\frac{t^{\alpha }}{\Gamma (\alpha +1)}+\vartheta_{2}\left(x\right)\frac{t^{2\alpha }}{\Gamma (2\alpha +1)}\right)}^{2}\}]. \end{array}$$

After the simplifications, we can obtain such as

$$\begin{array}{c} \text{Res}\;\vartheta_{3}\left(x,t\right)=\vartheta_{3}\left(x\right)\frac{t^{3\alpha }}{\Gamma (3\alpha +1)}-\frac{25e^{\frac{x}{\sqrt{6}}}\left(5-15e^{\sqrt{\frac{2}{3}}x}+20e^{\sqrt{\frac{3}{2}}x}-6e^{\frac{x}{\sqrt{6}}}\right)}{108{\left(1+e^{\frac{x}{\sqrt{6}}}\right)}^{6}}\frac{t^{3\alpha }}{\Gamma (3\alpha +1)}, \end{array}$$ (51)

Since Res *ϑ*_3_(*x*, *t*) ∣_*t*=0_ = 0, thus Eq (51) yields

$$\begin{array}{c} \vartheta_{3}\left(x\right)=\frac{25e^{\frac{x}{\sqrt{6}}}\left(5-15e^{\sqrt{\frac{2}{3}}x}+20e^{\sqrt{\frac{3}{2}}x}-6e^{\frac{x}{\sqrt{6}}}\right)}{108{\left(1+e^{\frac{x}{\sqrt{6}}}\right)}^{6}}. \end{array}$$ (52)

Proceeding in the similar process, we can obtain the Eq (37) such as

$$\begin{array}{c} \begin{array}{cc} \vartheta (x,t)= & \vartheta_{0}\left(x\right)+\vartheta_{1}\left(x\right)\frac{t^{\alpha }}{\Gamma (\alpha +1)}+\vartheta_{2}\left(x\right)\frac{t^{2\alpha }}{\Gamma (2\alpha +1)}+\vartheta_{3}\left(x\right)\frac{t^{3\alpha }}{\Gamma (3\alpha +1)}+\cdots ,\\ = & \frac{1}{{\left(1+e^{\frac{x}{\sqrt{6}}}\right)}^{2}}+\frac{5e^{\frac{x}{\sqrt{6}}}}{3{\left(1+e^{\frac{x}{\sqrt{6}}}\right)}^{3}}\frac{t^{\alpha }}{\Gamma (\alpha +1)}+\frac{25e^{\frac{x}{\sqrt{6}}}\left(-1+2e^{\frac{x}{\sqrt{6}}}\right)}{18{\left(1+e^{\frac{x}{\sqrt{6}}}\right)}^{4}}\frac{t^{2\alpha }}{\Gamma (2\alpha +1)}\\ & +\frac{25e^{\frac{x}{\sqrt{6}}}\left(5-15e^{\sqrt{\frac{2}{3}}x}+20e^{\sqrt{\frac{3}{2}}x}-18e^{\frac{x}{\sqrt{6}}}\right)}{108{\left(1+e^{\frac{x}{\sqrt{6}}}\right)}^{6}}\frac{t^{3\alpha }}{\Gamma (3\alpha +1)}+\cdots . \end{array} \end{array}$$ (53)

The obtained series moves towards the exact solution at *α* = 1 as follows

$$\begin{array}{c} \vartheta \left(x,t\right)=\frac{1}{{\left(1+e^{\frac{x}{\sqrt{6}}-\frac{5}{6}t}\right)}^{2}}. \end{array}$$ (54)

**Fig 3:**
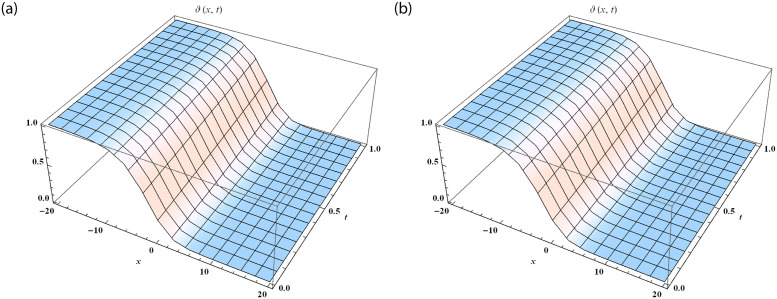
The 3D comparison of fractional NWS model with the SRPSM solution at *α* = 1 and the exact solution. (**a**) The approximate solution of *ϑ*_3_(*x*, *t*). (**b**) The exact solution of *ϑ*(*x*, *t*).

**Fig 4:**
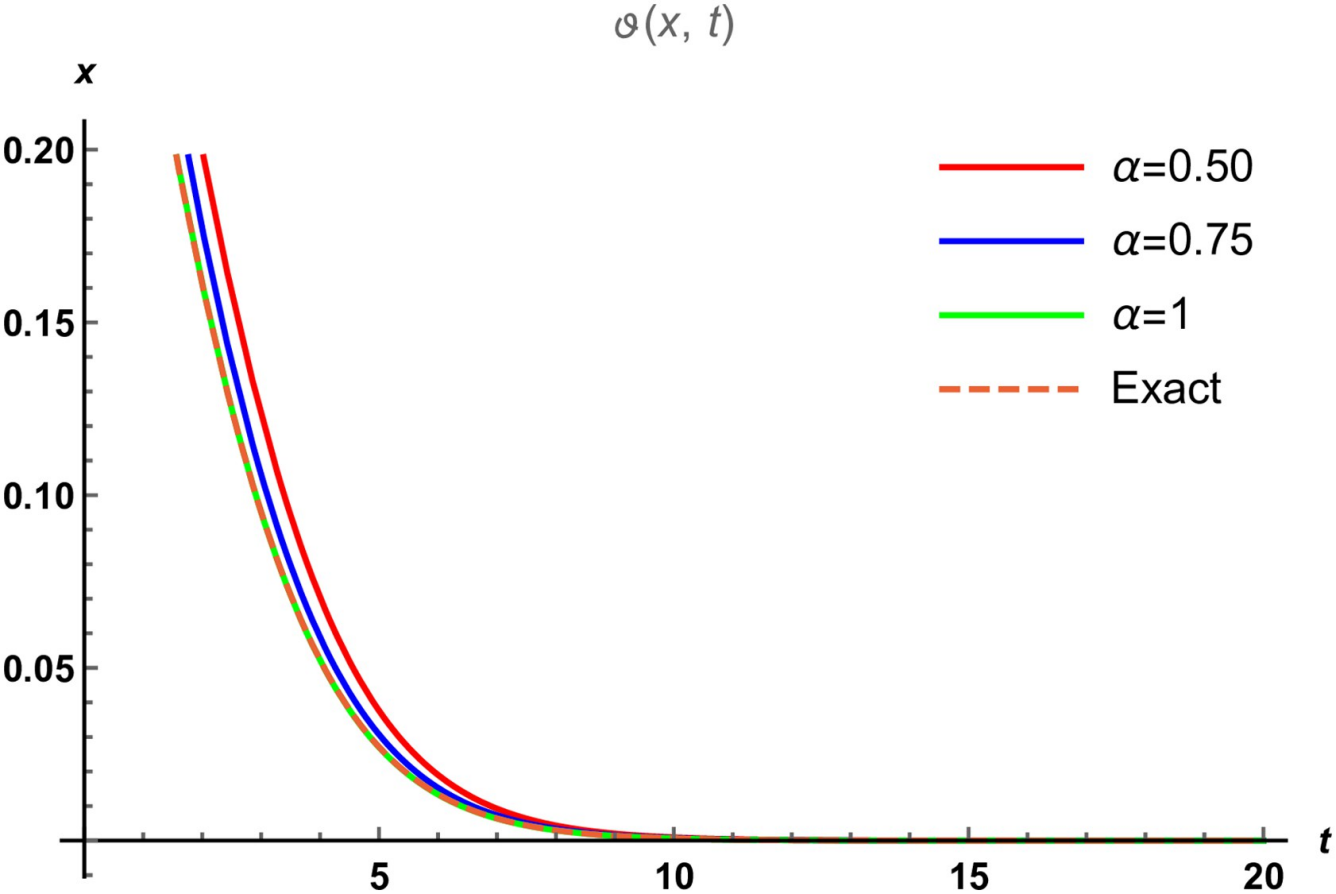
The physical nature of fractional NWS model compared with the exact solution and the SRPSM solution at various fractional order where *α* ∈ [0.5, 0.75, 1].

We divide the figures into two parts such that Fig 3a is designed by the solution of SRPSM at *α* = 1 and Fig 3b is designed by the exact solution of fractional NWS model. The range of space variable is taken as −20 ≤ *x* ≤ 20 at *t* = 1 for both 3D graphical solutions of Eqs (53) and (54). Fig 4 represents the physical nature of the fractional NWS model by considering the range of space values 0 ≤ *x* ≤ 20 and *t* = 0.5 at the various fractional order of *α* = 0.5, 0.75, 1 and then compared with the exact solution. We and then compared with the exact solution. We observe that the approximate solution derived by SRPSM converges to fractional order values. We consider our iteration only up to 3rd term and for better results these iterations can be extended. We also provide the absolute error in Table 2 to show that the approximate solutions obtained by our proposed strategy have excellent agreement with the exact solution. We provide these values at *α* = 0.50 and 1 to show that the obtained results become closer to the precise results as the fractional order raises. The graphical structures and the absolute error display that our proposed strategy is valid and accurate.

**Table 2:** The error distribution between the results of SRPSM and the exact solution.

*x*	*t*	*ϑ*_3_(*x*, *t*) at *α* = 0.5	*ϑ*_3_(*x*, *t*) at *α* = 1	Exact solution *ϑ*(*x*, *t*)	Absolute Error |*ϑ*(*x*, *t*)−*ϑ*_3_(*x*, *t*)|
0.25	0.05	0.275819	0.235022	0.235134	0.0001
	0.15	0.31605	0.254886	0.255767	0.0012
	0.25	0.346558	0.275149	0.277247	0.002
	0.35	0.373602	0.296077	0.2994993	0.003
	0.45	0.398923	0.317937	0.322413	0.005
0.5	0.05	0.249425	0.210998	0.2111117	0.001
	0.15	0.287675	0.229679	0.23062	0.001
	0.25	0.316991	0.248804	0.251053	0.003
	0.35	0.343204	0.268668	0.27235	0.004
	0.45	0.367925	0.289567	0.294431	0.005
0.75	0.05	0.224583	0.188452	0.188576	0.0001
	0.15	0.26157	0.205886	0.206868	0.001
	0.25	0.2908	0.223799	0.226154	0.002
	0.35	0.317572	0.242511	0.246383	0.003
	0.45	0.343316	0.262342	0.267492	0.005
1	0.05	0.201082	0.167455	0.167582	0.0001
	0.15	0.236176	0.183602	0.184606	0.001
	0.25	0.264473	0.200255	0.202668	0.002
	0.35	0.290781	0.21775	0.221735	0.004
	0.45	0.316379	0.236813	0.241758	0.004

## 5 Conclusion

This work presents the approximate solution of the fractional Newell-Whitehead-Segel model using the Sumudu residual power series method (SRPSM). The fractional order is considered in the Caputo sense. ST can break the ST can break the fractional order and generate a recurrence model. Now, RPSM is the best approach to handle this recurrence model and can generate the series solution. Additionally, RPSM can overcome the nonlinear terms that lead to simple iterations. We obtain the absolute error by using the difference of the precise results with the SRPSM authenticity of the proposed scheme. The 2D and 3D plot distribution reveals that our developed scheme has excellent agreement toward the exact solution. In the future, we aim to implement this scheme for other fractional order models in science and engineering.
